# Every-other-day fasting inhibits pyroptosis while regulating bile acid metabolism and activating TGR5 signaling in spinal cord injury

**DOI:** 10.3389/fnmol.2024.1466125

**Published:** 2024-09-12

**Authors:** Honghu Song, Rizhao Pang, Zhixuan Chen, Linjie Wang, Xiaomin Hu, Jingzhi Feng, Wenchun Wang, Jiancheng Liu, Anren Zhang

**Affiliations:** ^1^School of Health Preservation and Rehabilitation, Chengdu University of Traditional Chinese Medicine, Chengdu, China; ^2^Department of Rehabilitation Medicine, General Hospital of Western Theater Command, Chengdu, China; ^3^Department of Rehabilitation Medicine, Shanghai Fourth People's Hospital Affiliated to Tongji University, Shanghai, China

**Keywords:** every-other-day fasting, spinal cord injury, trans-membrane G protein-coupled receptor 5, NLRP3/Caspase-1/GSDMD pathway, pyroptosis

## Abstract

Every-other-day fasting (EODF) is a form of caloric restriction that alternates between periods of normal eating and fasting, aimed at preventing and treating diseases. This approach has gained widespread usage in basic research on neurological conditions, including spinal cord injury, and has demonstrated significant neuroprotective effects. Additionally, EODF is noted for its safety and feasibility, suggesting broad potential for application. This study aims to evaluate the therapeutic effects of EODF on spinal cord injury and to investigate and enhance its underlying mechanisms. Initially, the SCI rat model was utilized to evaluate the effects of EODF on pathological injury and motor function. Subsequently, considering the enhancement of metabolism through EODF, bile acid metabolism in SCI rats was analyzed using liquid chromatography-mass spectrometry (LC–MS), and the expression of the bile acid receptor TGR5 was further assessed. Ultimately, it was confirmed that EODF influences the activation of microglia and NLRP3 inflammasomes associated with the TGR5 signaling, along with the expression of downstream pyroptosis pathway related proteins and inflammatory cytokines, as evidenced by the activation of the NLRP3/Caspase-1/GSDMD pyroptosis pathway in SCI rats. The results demonstrated that EODF significantly enhanced the recovery of motor function and reduced pathological damage in SCI rats while controlling weight gain. Notably, EODF promoted the secretion of bile acid metabolites, activated TGR5, and inhibited the NLRP3/Caspase-1/GSDMD pyroptosis pathway and inflammation in these rats. In summary, EODF could mitigate secondary injury after SCI and foster functional recovery by improving metabolism, activating the TGR5 signaling and inhibiting the NLRP3 pyroptosis pathway.

## 1 Introduction

Spinal cord injury (SCI) is a severe traumatic condition causing either temporary or permanent dysfunctions in motor, sensory, and autonomic systems, leading to disability and reduced quality of life. Annually, there are an estimated 0.76 million new cases of traumatic spinal cord injury globally (Kumar et al., 2018), with the severity of injury varying among patients (Dimitrijevic and Kakulas, 2020). Nevertheless, current clinical treatments for SCI are limited and generally ineffective, imposing a significant burden on public health, labor costs, and the global economy. Given the irreversibility of primary injuries, understanding the pathological changes and progression post-SCI, and researching methods to prevent or mitigate secondary injuries and complications, have become critical areas of focus in SCI research.

Pyroptosis, also referred to as inflammatory necrosis of cells, is a form of programmed cell death triggered by inflammasomes. The NLRP3/Caspase-1/GSDMD pathway represents a classic mechanism of cell pyroptosis. Specifically, the activation of the NOD-like receptor protein 3 (NLRP3) leads to the activation of cysteinyl aspartate specific proteinase-1 (Caspase-1), which subsequently induces the cleavage of gasdermin D (GSDMD). This process results in the maturation and secretion of pro-inflammatory cytokines IL-18 and IL-1β, mediating pyroptosis and causing cell swelling, ultimately leading to cell lysis and death (Shi et al., 2015; Zhang et al., 2021). Pyroptosis plays a crucial role in secondary injury following SCI. On the one hand, SCI activates NLRP3 inflammasomes, triggering the pyroptosis pathway and leading to cell death. On the other hand, pyroptosis can intensify the inflammatory response and neuronal loss associated with SCI. Therefore, inhibiting NLRP3 inflammasomes and cellular pyroptosis offers an effective treatment strategy for SCI (Jiang et al., 2023; Liu et al., 2023).

Research has confirmed that bile acids (BAs) play an important role in the central nervous system (Monteiro-Cardoso et al., 2021) and can be utilized to treat neurological diseases (Hurley et al., 2022). Evidence indicates that bile acid administration can alleviate secondary injuries post-SCI and enhance functional recovery (Kim et al., 2018; Ko et al., 2019; Hou et al., 2021). However, the precise mechanisms through which BAs exert therapeutic effects in SCI remain largely unexplored. The trans-membrane G protein-coupled receptor 5 (TGR5), a bile acid receptor extensively distributed and expressed in the central nervous system (Duboc et al., 2014), can be activated by bile acids. Previous studies have shown that TGR5 activation could inhibit NLRP3 and reduce neuroinflammation (Liang et al., 2021). Additionally, Zhang et al. (2024) pointed out that the bile acid UDCA influences NLRP3 inflammasome-mediated neuroinflammation in middle cerebral ischemia/reperfusion occlusion (MCAO) mice through TGR5 regulation. These findings suggest that TGR5 could be an effective therapeutic target for neurological diseases. While the influence of TGR5 on NLRP3 inflammasomes highlights its potential for treating neuroinflammation, further investigation is needed to understand the effects of TGR5 activation on NLRP3 inflammasome-mediated pyroptosis and the subsequent inflammatory response following SCI.

Intermittent fasting (IF) and metabolic switching could optimize physiological functions, delay aging, and enhance neuroplasticity and resistance of the brain to injury and disease (Anton et al., 2018; Mattson et al., 2018). Every-other-day fasting (EODF) is a type of IF that alternates between normal diet and fasting, emerging as a novel approach to prevent and treat certain diseases. It has been employed in foundational research on various neurological conditions such as stroke and traumatic brain injury and has demonstrated significant neuroprotective effects (Fann et al., 2014; Mattson et al., 2018; Hadem et al., 2019; Rubovitch et al., 2019). Furthermore, it is worth noting that IF could inhibit the activation of pro-inflammatory microglia after intracerebral hemorrhage (Dai et al., 2022), which is related to NLRP3 inflammasome assembly and caspase-1 activation (Ghosh et al., 2018; Dai et al., 2019), highlighting the potential of EODF in restraining the NLRP3 pyroptosis pathway. Recently, an increasing number of studies have investigated EODF’s application to spinal cord injury (SCI), including its ability to inhibit disease progression, reduce cell apoptosis, and promote nerve fiber repair (Sayadi et al., 2021; Li et al., 2022), thus confirming its therapeutic potential for SCI. However, the exact mechanism by which EODF provides its therapeutic benefits remains elusive. It has been reported that intermittent fasting can modulate the composition of gut microbiota and influence metabolism (Patterson and Sears, 2017; Frank et al., 2021). Additionally, recent research has demonstrated that intermittent fasting can effectively regulate bile acid metabolism in non-alcoholic steatohepatitis mice (Lin et al., 2023). Consequently, it is plausible that EODF intervention may exert therapeutic effects after SCI by regulating bile acid metabolism and inhibiting the progression of secondary injury.

In this study, we aim to use liquid chromatography-mass spectrometry (LC–MS) to analyze changes in bile acid metabolism and investigate whether EODF intervention (alternating 24-h periods of unrestricted eating and fasting, sustained until the end of the study) can regulate bile acid metabolism following SCI. Additionally, we explore whether this intervention can alleviate pyroptosis and neuroinflammation post-SCI by activating TGR5 signaling and inhibiting NLRP3 inflammasomes.

## 2 Materials and methods

### 2.1 Animals and SCI model

The procedures in this study were approved by the Ethics Committee for Animal Experiments of the General Hospital of Western Theater Command. Thirty healthy male Sprague–Dawley rats, each weighing between 270 and 300 g, were acquired from SiPeiFu (Beijing) Biotechnology Co., Ltd. (Animal Certificate No.: SCXK (Beijing) 2019–0010) and housed in standard plastic cages (480 × 350 × 200 mm, 3 ~ 4 animals per cage) in a quiet and clean animal room with a controlled temperature of 22 ± 2°C and an artificial 12-h light–dark cycle (lights on at 8: 00 a.m.).

**Figure 1:**
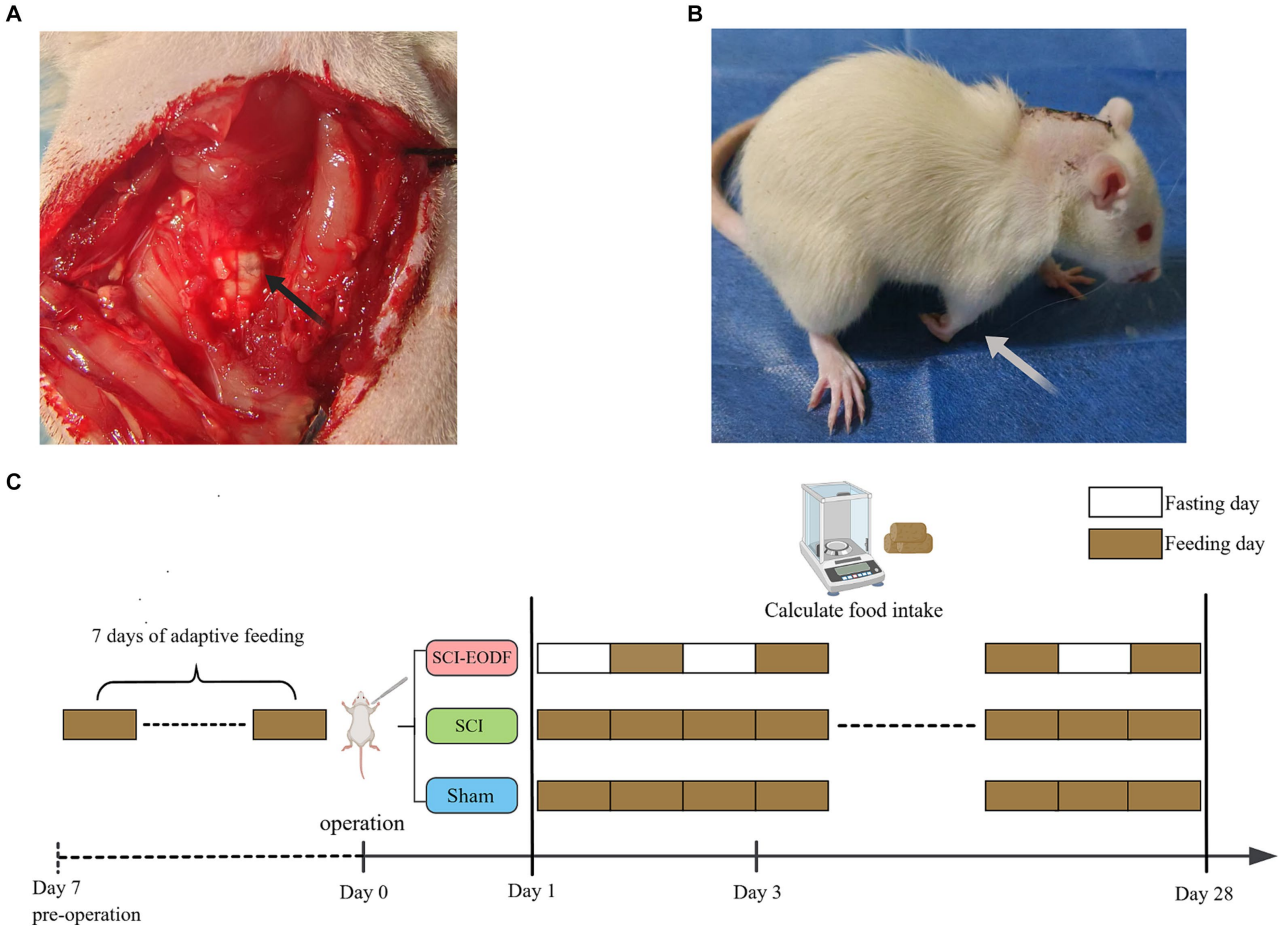
Establishment of SCI animal model and EODF intervention. **(A)** C5 laminectomy and spinal cord exposure. **(B)** Rats that underwent SCI surgery showed functional impairment in the affected forelimb. **(C)** After the molding was completed, rats in both the Sham and SCI groups were given unrestricted access to food and water, whereas the SCI_EODF group commenced the EODF diet immediately post-surgery. The EODF diet involves alternating 24-h periods of fasting and unrestricted eating, with water available at all times.

Changes in the environment could affect rats to varying degrees, potentially skewing subsequent experimental results. Therefore, rats should undergo a 7-day adaptive feeding period to fully adjust to their surroundings. Following this period, 30 rats were randomly divided into three groups: the Sham group, the SCI group, and the EODF intervention group (SCI_EODF). In the SCI and SCI_EODF groups, 20 rats underwent surgery to establish a C5 hemi-clamp spinal cord injury model. Anesthesia was induced using an intraperitoneal injection of 5% pentobarbital sodium at a dosage of 30 mg/kg. Subsequent to the C5 laminectomy, the spinal cord was clamped at the corresponding level for 30 s using 70 g medical aneurysm clips (Figure 1A). To prevent dehydration, 5 mL of normal saline was injected intraperitoneally after suturing and continued for 3 days post-surgery. The successful indicators of the model include the injured forelimb of the rat adopting a club-like shape post-surgery; when walking, the rat’s body deviated toward the injured side. Additionally, when lifting the rat by its tail and suspending it in the air, the affected forelimb failed to extend forward and downward (Figure 1B). The procedure for the 10 rats in the sham surgery group was limited to C5 laminectomy, ensuring the spinal cord remained intact.

After the molding was completed, rats in both the Sham and SCI groups were given unrestricted access to food and water, whereas the SCI_EODF group commenced the EODF diet immediately post-surgery. The EODF diet involves alternating 24-h periods of fasting and unrestricted eating, with water available at all times. On fasting days, food is removed at 6 pm and returned at 6 pm the following day, initiating a cycle of free feeding until 6 pm on the subsequent day. This pattern continues until the experiment concludes. The intervention lasted for 28 days (Figure 1C).

### 2.2 Animal feeding and body weight monitoring

Preweighed food was placed in each cage’s funnel at 6 p.m. every day, with the remaining amount measured after 24 h. Subsequently, the rats were either provided with fresh food or fasted for the next 24 h depending on group assignments. The average daily food intake per group was calculated by dividing the total weekly consumption by seven. Concurrently, rats were weighed prior to the injury and weekly thereafter until the study concluded.

### 2.3 Behavioral evaluation and motor function assessment

**Figure 2:**
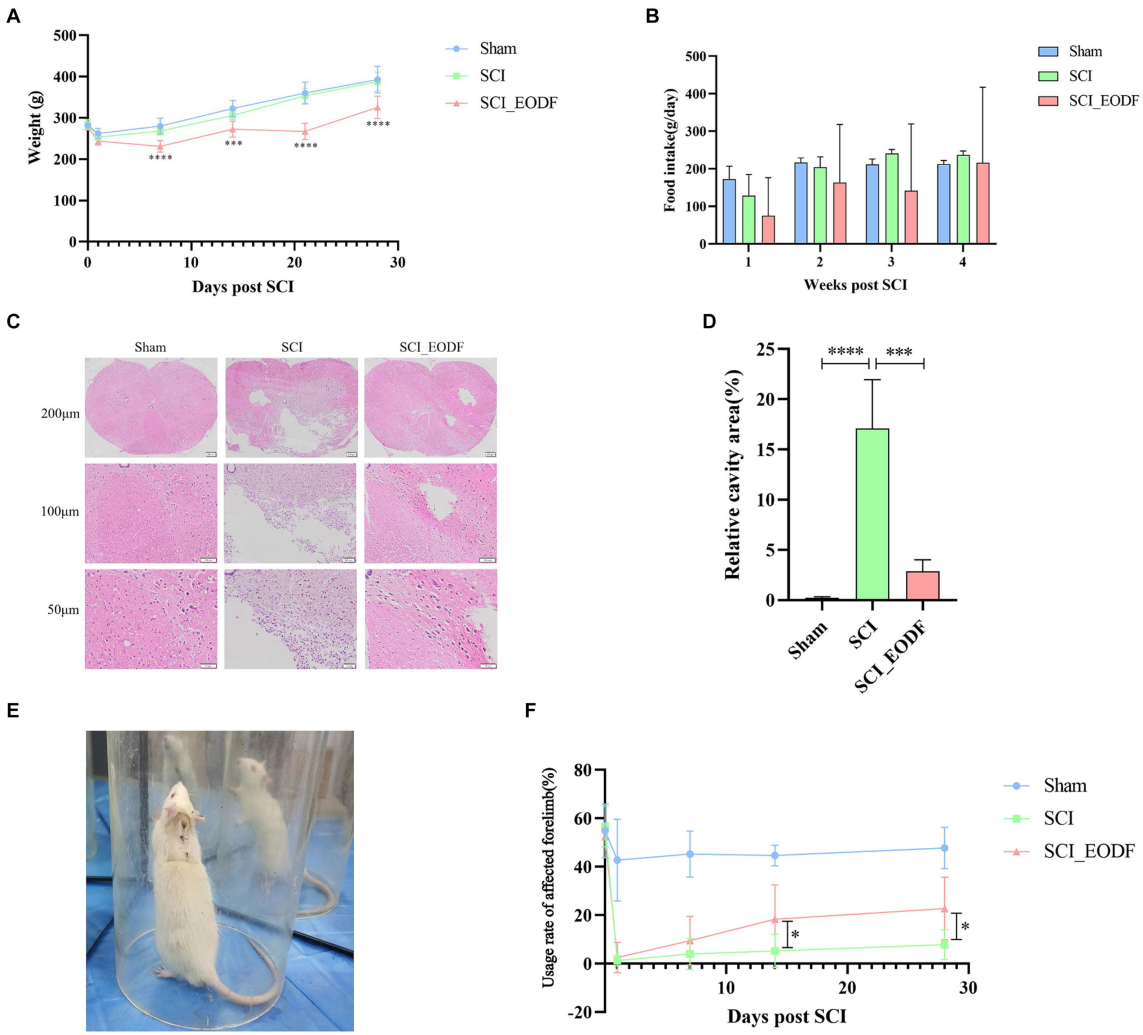
Evaluation of general condition, motor function, and pathological injury recovery in SCI rats. **(A)** Weight of rats up to 28 days after operation. **(B)** Food intake of rats up to 28 days after operation. **(C)** Analysis of pathological injury recovery of rat spinal cord tissues by HE staining. **(D)** Quantitative analysis of the relative cavity area in spinal cord tissue across different rat groups. **(E)** Each group of rats was placed in transparent cylinders for Cylinder Rearing test. **(F)** Comparative analysis of the usage rate of the affected forelimb in rats up to 28 days post-operation using one-way ANOVA. The usage rate of the affected forelimb was calculated as: the total number of times the affected forelimb collided with the wall/(the number of times the affected forelimb collided independently + the number of times the contralateral forelimb collided independently + the number of times both forelimbs collided together). Data are presented as mean ± SD. *p < 0.05, ***p < 0.001, ****p < 0.0001.

Behavioral evaluations were conducted on each group of rats before surgery, and at 1, 7, 14, and 28 days post-surgery. Specifically, the recovery of motor function in the affected forelimbs was assessed using the Cylinder Rearing test, a cost-effective and convenient method widely used for evaluating forelimb movement deficits in rats (Roome and Vanderluit, 2015), which determines the recovery of motor function by calculating the usage rate of the affected forelimb. More narrowly, the usage rate of the affected forelimb is defined as the total number of times the affected forelimb contacts the wall/ (the number of times the affected forelimb independently contacts the wall + the number of times the contralateral forelimb independently contacts the wall + the number of times both forelimbs contact the wall together). The total number of times the affected forelimb contacts the wall is calculated as the number of times the affected forelimb independently contacts the wall +1/2 × the number of times both forelimbs contact the wall together. A transparent cylinder (20 cm in diameter, 30 cm in height) was placed on a clean tabletop, and each rat was positioned inside. Specially designed mirrors were placed on both sides of the cylinder to observe the rats’ activity from various angles (Figure 2E). A camera, fixed on a tripod in front of the cylinder, recorded about 15 min of video per rat. All videos were evaluated by independent experimenters who were proficient in rating criteria and blinded to animal grouping.

### 2.4 HE staining

Spinal cord tissue obtained through perfusion was dehydrated and embedded in paraffin, then sectioned into 4 mm slices and dewaxed to water. The slices were stained as follows: (1) Soaked in hematoxylin for 5 min; (2) Differentiated in the differentiation solution for 30 s, quickly inserted and removed the slices; (3) Soaked in bluing solution for about 1 min; (4) Stained with eosin for 30 s to 1 min. Each step was followed by washing the slices with tap water. Finally, the slices underwent gradient ethanol dehydration from low to high concentrations and were subjected to standard clarification and sealing procedures. Upon completion, independent researchers outside the experimental group examined the images using a pathological microscope equipped with image acquisition systems. The pathological conditions of the spinal cord tissue were observed and documented at different magnifications. Finally, the size of the spinal cord tissue cavity area was quantified using Image J software based on the collected images.

### 2.5 Sample preparation for BA profiling and UPLC–MS/MS analysis

Serum Samples were extracted in 600 μL of methanol (−20°C) with 100 mg of glass beads, vortex for 60 s. Centrifuge at 12,000 rpm and 4°C for 10 min, take 400 μL of the supernatant and concentrate it to dryness with a vacuum concentrator. Add 100 μL of 30% methanol to reconstitute. The supernatant was filtered through 0.22 μm membrane, and the filtrate was added to the LC–MS bottle.

The LC–MS analysis was performed on EXion LC Liquid chromatography (AB SCIEX, United States) and Mass spectrometric detection of metabolites was performed on AB6500 Plus (AB SCIEX, United States). ACQUITY UPLC^®^ BEH C18 column (2.1 × 100 mm, 1.7 μm, Waters, United States) was used, the injection volume was 5 μL, the column temperature was 40°C, and the mobile phase was A-0.01% formic acid water, B-acetonitrile. The gradient elution conditions were 0 ~ 4 min, 25% B; 4 ~ 9 min, 25 ~ 30% B; 9 ~ 14 min, 30 ~ 36% B; 14 ~ 18 min, 36 ~ 38% B; 18 ~ 24 min, 38 ~ 50% B; 24 ~ 32 min, 50 ~ 75% B; 32 ~ 33 min, 75 ~ 90% B; 33 ~ 35.5 min, 90 ~ 25% B. The flow rate was 0.25 mL/min.

The Mass spectrum is Electrospray ionization (ESI) source, negative ionization mode. The ion source temperature was 500°C, the ion source voltage was −4,500 V, the collision gas was 6 psi, the curtain gas was 30 psi, and the atomizing gas and auxiliary gas were both 50 psi. Scans were performed using multiple reaction monitoring (MRM).

### 2.6 Western blot

Total protein from spinal cord tissues was extracted using a protein extraction kit (Solarbio, Beijing, China) according to the provided instructions, and protein concentration was quantified using the BCA method. The samples were loaded in equal amounts of total protein following gel preparation. The proteins were separated by 10% SDS-PAGE (80 V for 40 min, then 160 V for 1 h) and transferred to a PVDF membrane. The membrane was blocked with 5% milk for 1 h and then incubated overnight at 4°C with primary antibodies against TGR5 (1:1,000, DF14067, Affinity Biosciences), NLRP3 (1:1,000, ab263899, Abcam), Caspase-1 (1:1,000, ab179515, Abcam), GSDMD (1:1,000, ab219800, Abcam), TMEM119 (1:20,000, 66,948-1-Ig, Proteintech), Vinculin (1:5,000, 26,520-1-AP, Proteintech), and GAPDH (1:5,000, 10,494-1-AP, Proteintech). This was succeeded by incubation with HRP-conjugated secondary antibodies (1:5,000, SA00001-2, Proteintech) for 1 h at room temperature. Finally, the membranes were visualized using ECL reagents and the Azure Biosystems NIR Fluorescence Imaging system, with quantitative grayscale analysis performed using ImageJ software.

### 2.7 Measurement of inflammatory cytokine concentrations

The serum levels of IL-18 and IL-1β in rats were measured using ELISA kits (Elabscience), adhering strictly to the manufacturer’s instructions. Standard curves were generated from the OD and concentration values of the standard samples to accurately quantify the concentration of each sample.

### 2.8 Statistical analysis

Statistical analysis was conducted using SPSS software version 25.0 (SPSS, Chicago, IL, United States). Data was evaluated for normal distribution. Comparative analysis among multiple groups utilized one-way ANOVA, followed by the least significant difference (LSD) test for pairwise comparisons, or nonparametric methods like the Kruskal-Wallis test and Mann–Whitney test. Repeated measures ANOVA was used in comparative analysis among multiple groups at different time points. A *p*-value of less than 0.05 was considered statistically significant.

## 3 Results

### 3.1 EODF intervention controls the weight and food intake of rats

Rats were weighed before injury and weekly thereafter. The daily food intake of each group per week was also accurately recorded (Figures 2A,B). There were no significant differences in average body weight among the Sham, SCI, and SCI_EODF groups before the surgery. On the first day post-surgery, the weights of all three groups visibly decreased, indicating that SCI could transiently reduce rat weight, potentially due to decreased food intake following injury. From the first day after surgery, all groups exhibited a weight gain trend. However, the SCI_EODF group consistently showed lower body weight compared to the SCI group at all-time points, with statistically significant differences observed from day 7 onwards (*p* < 0.0001), suggesting that EODF effectively controls rat weight.

After SCI surgery, the average food intake of rats in the SCI and SCI_EODF groups decreased in the first week compared to the Sham group, which indicates that SCI could affect the food intake of rats. Over time, the daily average food intake in each group showed an upward trend and gradually stabilized. Although the overall average food intake of SCI_EODF rats remained consistently lower than that of the SCI and Sham groups at all observed time points after surgery, their food intake on feeding days partially compensated for the zero intake on fasting days, reaching 91% of the SCI group’s intake by the fourth week. These findings indicate that the EODF intervention primarily improves metabolism and effectively controls body weight in rats by regulating their food intake rhythm rather than the quantity consumed, without causing malnutrition.

### 3.2 EODF promotes pathological injury recovery and improves motor function in SCI rats

HE staining was employed to examine the pathological changes in spinal cord tissues of each rat group (Figure 2C). The results showed that the spinal cord tissues in the Sham group remained normal with distinct boundaries, devoid of any pathological changes such as bleeding, inflammatory cell infiltration, or necrosis. In contrast, the SCI group exhibited significant pathological damage with large cavities in the spinal cord tissues and minimal recovery. The SCI_EODF group, however, demonstrated better recovery, showing only small vacuoles and clear boundaries. The morphology and structure in this group were more similar to those in the Sham group. Furthermore, Image J software was employed to quantitatively analyze the percentage of cavity area in the spinal cord tissues of each group of rats (Figure 2D). The results revealed that the Sham group had a significantly lower cavity area percentage compared to the other two groups. Notably, the relative cavity area in the spinal cord tissues of the SCI_EODF group was significantly reduced compared to the SCI group (*p* < 0.001). The above results suggest that EODF intervention could alleviate spinal cord pathological damage in SCI rats.

The findings from the Cylinder Rearing test further confirmed that EODF intervention aids in rehabilitation and enhances motor function in SCI rats. Prior to SCI surgery, there were no significant differences in the usage rate of the affected forelimbs among the different groups (*p* > 0.05). One day after surgery, a slight decrease in the utilization rate of the affected forelimbs was observed in the Sham group, whereas the other two groups experienced a significant decline, which gradually improved over time. Throughout the experiment, the Sham group consistently exhibited higher utilization rates of the injured forelimbs compared to the other groups. Notably, the SCI_EODF group demonstrated superior recovery at all postoperative time points compared to the SCI group, and one-way ANOVA showed statistically significant differences between the two groups on days 14 and 28 (*p* < 0.05) (Figure 2F). Although no statistical significance was observed through two-way repeated measures ANOVA (*p* = 0.0843, Mean Diff. = 14.96, DF = 7.151), both the main effects of time and grouping, as well as their interaction, significantly influenced the recovery of motor function in rats (F _time_ = 50.99, F _grouping_ = 87.57, F _interaction_ = 7.483, *p* < 0.0001).

### 3.3 The serum BAs levels are increased in SCI_EODF rats

**Figure 3:**
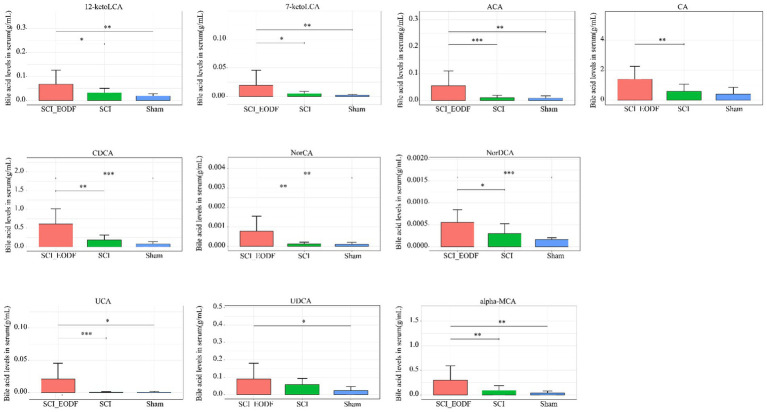
The UPLC-MS/MS analysis and profiling of bile acid metabolites in rat serum on Day 28 revealed a significant increase in various bile acids in the SCI_EODF group. Elevated levels included 12-ketoLCA, 7-ketoLCA, ACA, CA, CDCA, NorCA, NorDCA, UCA, UDCA and alpha-MCA. Data are presented as mean ± SD. *p < 0.05, **p < 0.01, ***p < 0.001.

To investigate the changes in bile acid metabolites following SCI and EODF intervention, we collected serum from rats in the Sham, SCI, and SCI_EODF groups and sacrificed the rats on day 28. Our findings revealed that the levels of various bile acids in the serum of the SCI_EODF group were higher than those in the Sham and SCI groups (Figure 3). In contrast, there was no significant difference between the Sham and SCI groups. These results indicate that BA metabolites were elevated in SCI rats following EODF intervention, aligning with previous studies that report fasting can regulate bile acid metabolism (Lin et al., 2023; Gregor et al., 2024).

### 3.4 EODF activates TGR5 signaling and inhibits NLRP3/Caspase-1/GSDMD pathway to prevent pyroptosis

**Figure 4:**
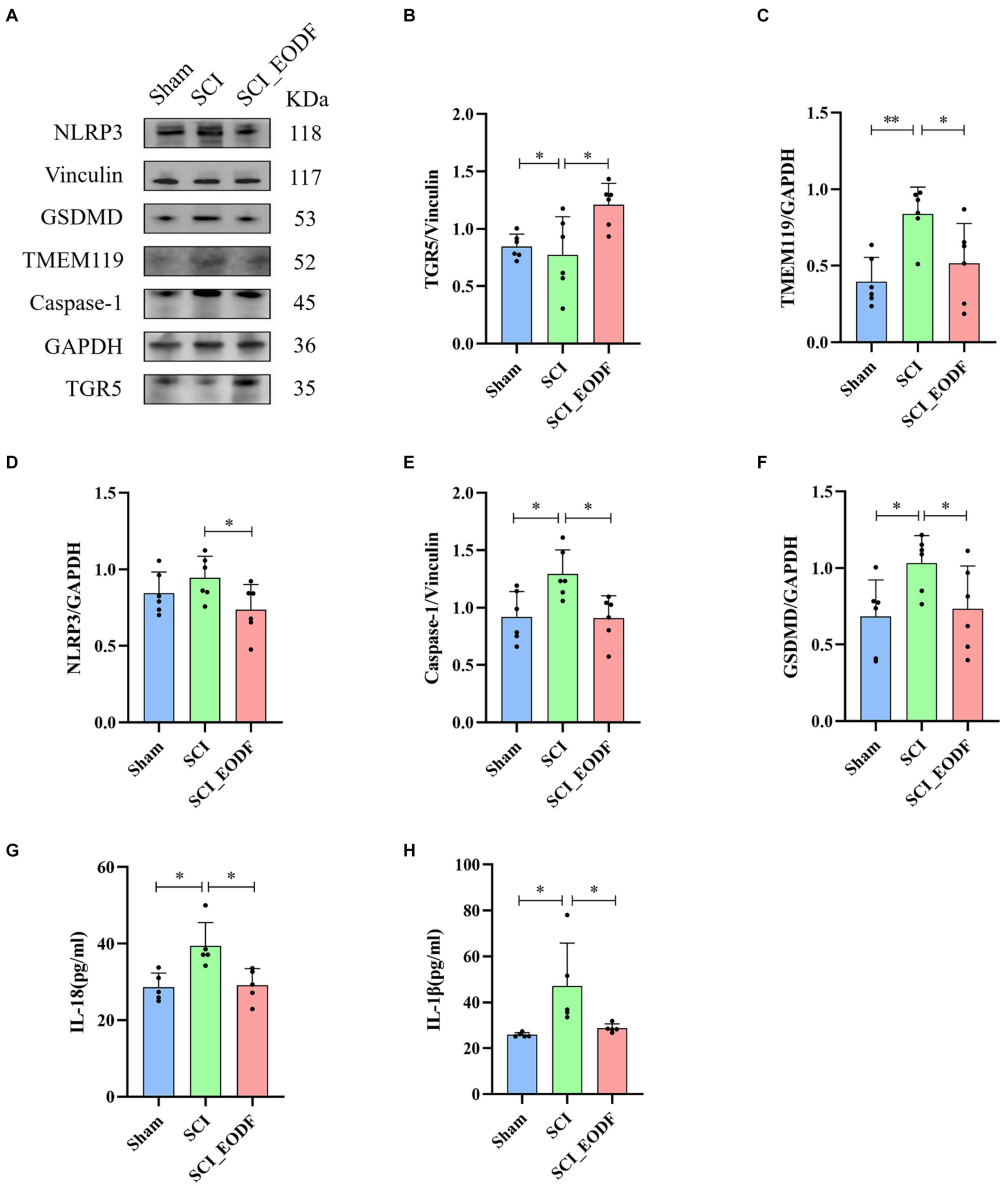
EODF inhibits the NLRP3/Caspase-1/GSDMD pyroptosis pathway and reduce the levels of inflammatory cytokines IL-18 and IL-1β downstream of the pyroptosis pathway while activating TGR5 and suppressing microglia activation. **(A–F)** Western blotting analysis of TGR5, TMEM119, NLRP3, Caspase-1 and GSDMD expression in spinal cord tissues. **(G,H)** The levels of inflammatory cytokines IL-18 and IL-1β evaluated by ELISA in three groups of rats. Data are presented as mean ± SD. *p < 0.05, **p < 0.01.

Bile acids are known to upregulate the expression of various cell receptors, particularly TGR5. Observing a significant increase in serum BAs levels following SCI after EODF intervention, we further examined TGR5 expression in the spinal cord and discovered a notable rise in TGR5 protein levels. Moreover, we noted a reduction in the expression of TMEM119, a marker typical of microglia. Activation of TGR5 and suppression of microglia have been shown to be associated with decreased expression of NLRP3 inflammasome, suggesting the potential of EODF to inhibit the NLRP3/Caspase-1/GSDMD pathway and control pyroptosis. Subsequent tests confirmed that EODF intervention and TGR5 activation indeed inhibited the expression of NLRP3 inflammasomes, Caspase-1, and GSDMD in SCI rats (Figures 4A–F), while also reducing the levels of inflammatory cytokines IL-18 and IL-1β downstream of the pyroptosis pathway in the rat serum (Figures 4G,H).

## 4 Discussion

**Figure 5:**
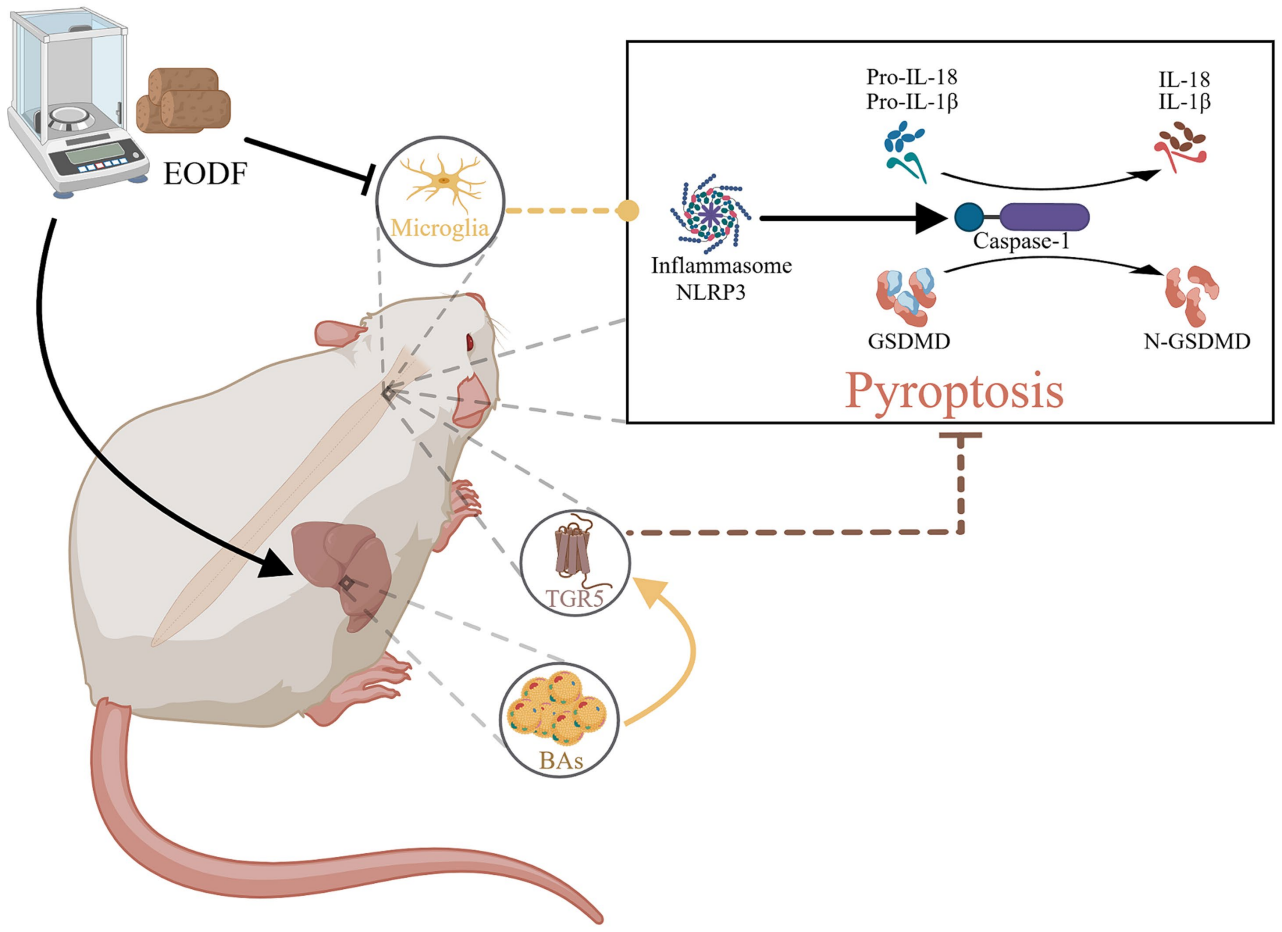
The therapeutic mechanism of EODF. EODF could inhibit the activation of microglia and enhance the secretion of various bile acids in SCI rats, which subsequently activate the bile acid receptor TGR5 in spinal cord tissue. This activation correlates with the suppression of the NLRP3/Caspase-1/GSDMD pyroptosis pathway induced by spinal cord injury, thus exerting neuroprotective effects.

Our study demonstrated that every-other-day fasting (EODF), as a simple and safe dietary intervention, can effectively control both weight and food intake in spinal cord injury (SCI) rats. Crucially, EODF intervention significantly reduces pathological damage post-SCI and enhances motor function. Further research revealed that EODF intervention not only inhibits microglial activation but also elevates serum bile acid levels, thereby increasing the expression of TGR5, which was associated with the inhibition of the NLRP3/Caspase-1/GSDMD pyroptosis pathway and the regulation of pro-inflammatory cytokines IL-18 and IL-1β (Figure 5).

EODF, an improved calorie restriction (CR) method, controls caloric intake without causing malnutrition and has the potential to enhance general health and prevent diseases (Mattson et al., 2017). Scientific research has validated the benefits of EODF, particularly its effective metabolic regulation. Harris et al. (2018) noted that the most direct effect of EODF is the reduction of obesity and overall weight. In this study, it was observed that although the food intake of SCI rats under EODF intervention gradually increased, their weight remained effectively controlled. Similarly, Panizza et al. (2019) suggested that intermittent fasting might be more effective in improving liver function than a healthful diet pattern. Our profiling of bile acid metabolites in rats also revealed elevated levels of various bile acids in the serum of SCI rats treated with EODF, indicating the role of EODF in enhancing liver metabolic function. More importantly, EODF has shown potential in treating neurological diseases. Early studies have demonstrated its ability to promote functional recovery after SCI (Plunet et al., 2008, 2010; Jeong et al., 2011), aligning with the findings of this study. Further investigation has revealed that mechanisms such as anti-inflammatory effects and inhibition of microglial activation (Fann et al., 2014; Vasconcelos et al., 2014; Dai et al., 2022) may underlie the neuroprotective benefits of EODF. Additionally, recent research indicates that EODF might aid in nerve regeneration and repair following nerve injury by modulating the secretion of various metabolites (Serger et al., 2022), thereby exerting anti-inflammatory effects (Pereira et al., 2024). Based on the potential neuroprotective effects of bile acids and our findings that EODF improves bile acid metabolism in SCI rats, we speculate that elevated bile acid metabolite levels may contribute to the neuroprotective mechanism of EODF.

Inflammasomes are large molecular platforms found in the cytoplasm, with the NOD-Like Receptors (NLRs) inflammasome family being the primary member, involved in innate and adaptive immune responses (Almeida-da-Silva et al., 2023; Tsankov et al., 2024). They also play a role in central nervous system diseases (Kong et al., 2017). Among them, the NLRP3 inflammasome is the most prevalent type within the NLR family (Ramachandran et al., 2024), mediating inflammatory responses across various diseases (Xu et al., 2024). It activates the classic Caspase-1 dependent pyroptosis pathway (Man et al., 2017), known as the NLRP3/Caspase-1/GSDMD pathway, which results in the release of inflammatory cytokines IL-1β and IL-18, thus amplifying the inflammatory response. Given the close association between the NLRP3 inflammasome, cell pyroptosis, and secondary injury post-SCI (Jiang et al., 2023; Liu et al., 2023), along with the regulatory effect of fasting on the NLRP3 inflammasome (Traba et al., 2015, 2017), it is suggested that the modulation of pyroptosis might be crucial for the therapeutic efficacy of EODF after SCI.

The trans-membrane G protein-coupled receptor 5 (TGR5) is widely distributed across various organs and can be activated by several bile acids, such as CA, CDCA, DCA, LCA, and UDCA (Smaling et al., 2023). As a member of the G protein-coupled receptors (GPCRs) family, TGR5 activation effectively regulates metabolism and controls obesity (Lun et al., 2024). With ongoing research, there are additional reports highlighting the role of TGR5 in immune regulation and inflammatory signaling (Ye et al., 2024). Furthermore, the activation of TGR5 can exert neuroprotective effects. For instance, administering bile acids like CA, CDCA, and UDCA promotes recovery in neurodegenerative diseases such as CTX, Alzheimer’s disease, and Parkinson’s disease (Monteiro-Cardoso et al., 2021). UDCA and TUDCA have also shown potential in treating spinal cord injury (Ko et al., 2019; Hou et al., 2021). Additionally, TGR5 activation can mitigate NLRP3 inflammasome-mediated neuroinflammation following subarachnoid hemorrhage (Hu et al., 2021). In ischemic stroke models, bile acid-induced TGR5 activation reduces the expression of NLRP3 inflammasomes and IL-1β (Zhang et al., 2024), offering therapeutic benefits. Notably, the regulatory effect of EODF on bile acid metabolism levels suggests its association with TGR5. Simultaneously, EODF can significantly enhance metabolism and control inflammation (Fann et al., 2014), akin to the effects of activating TGR5. This suggests that TGR5 signaling might be crucial in EODF’s regulation of pyroptosis following SCI. Nonetheless, the relationship between TGR5 activation, reduced pyroptosis, and EODF intervention after SCI remains incompletely understood. Our study discovered that EODF intervention following SCI boosts the production of various bile acids and activates the bile acid receptor TGR5. Concurrently, the activation of TGR5 was accompanied by the inhibition of NLRP3 inflammasomes, consistent with previous findings (Zhang et al., 2024). Correspondingly, EODF intervention also decreased the levels of key proteins in the NLRP3/Caspase-1/GSDMD pyroptosis signaling pathway and suppressed the expression of cytokines IL-1β and IL-18 in the SCI rat model.

Considering the substantial evidence from both basic and clinical studies underscoring the pivotal role of the NLRP3 pyroptosis signaling pathway in SCI pathology (Jiang et al., 2017; Dai et al., 2019; Xu et al., 2021), EODF intervention targeting this pathway, along with the activation of TGR5, presents a promising strategy for enhancing functional recovery after SCI.

The advantages of this study are as follows. On the one hand, it validated the therapeutic effects of EODF intervention on SCI rats and elucidated its mechanisms, suggesting that the NLRP3/Caspase-1/GSDMD pathway may play a role in the protective effects of EODF. On the other hand, from the perspective of EODF improving metabolism, we found that EODF can promote the production of bile acid metabolites, indicating that the activation of bile acid receptor TGR5 might be a crucial link between EODF and the NLRP3 pyroptosis pathway. Previous research has mainly focused on the therapeutic potential of drugs targeting TGR5 and NLRP3 inflammasomes. However, the EODF intervention in this study is a safe, non-invasive, and straightforward method. Its application in the clinical treatment of SCI could alleviate the economic burden on patients’ families and society, offering significant economic and social benefits. Furthermore, understanding the neuroprotective mechanisms of EODF can facilitate its clinical translational application and broader adoption. Despite the benefits of this study, several limitations remain. Firstly, while this experiment investigates the therapeutic mechanisms of spinal cord injury through the lens of pyroptosis and highlights a potential connection between TGR5 signaling and the NLRP3 pyroptosis pathway in EODF intervention, further studies are needed to elucidate the key roles of this G protein-coupled receptor. Secondly, although the study demonstrates that EODF can activate TGR5 by improving bile acid metabolism, the precise mechanism of this activation still needs further clarification. Lastly, the therapeutic mechanisms of EODF in SCI identified in this study need to be validated through clinical practice, and their effectiveness should be supported by additional clinical investigations.

In future research, we aim to further explore the therapeutic effects of EODF in spinal cord injury. To achieve this, the duration of the experiment will be carefully adjusted, incorporating multiple time points to compare the therapeutic effects of EODF interventions over various time spans and at different stages post-SCI. Meanwhile, it is necessary to identify potential targets of EODF to enhance its therapeutic mechanism. Additionally, the potential role of TGR5 warrants greater attention and investigation. Energy homeostasis and food intake are tightly regulated by various G protein-coupled receptors (GPCRs) expressed in central and peripheral cells. As a GPCR, the role of TGR5 in metabolism needs further study. We will also compare the therapeutic effects of EODF by creating additional experimental groups and using drugs targeting TGR5 to better elucidate the relationship between EODF and TGR5. Furthermore, gaining a deeper insight into the specific interactions between TGR5 and NLRP3, along with the various signaling pathways involving TGR5, is crucial for discovering new therapeutic strategies for SCI. Lastly, we will integrate relevant clinical trials to facilitate clinical applications of our findings.

## 5 Conclusion

In this study, we conducted animal experiments to validate the safety and efficacy of EODF intervention in treating SCI and to clarify the mechanisms by which EODF promotes spinal cord injury recovery. Our findings demonstrate that EODF improves metabolism, regulates weight, and suppresses microglial activation while activating TGR5 in relation to bile acid metabolism. What’s more, EODF could inhibit the NLRP3/Caspase-1/GSDMD pyroptosis pathway and reduce inflammatory responses post-SCI, thereby facilitating pathological repair of spinal cord tissue and enhancing motor function recovery. In summary, this study has the potential to provide substantial theoretical support and research foundation for the clinical application of EODF, as well as for the development of subsequent SCI treatment methods.

## Data Availability

The original contributions presented in the study are included in the article/Supplementary material, further inquiries can be directed to the corresponding authors.

## References

[ref1] Almeida-da-Silva C. L. C. Savio L. E. B. Coutinho-Silva R. Ojcius D. M. (2023). The role of NOD-like receptors in innate immunity. Front. Immunol. 14:1122586. doi: 10.3389/fimmu.2023.1122586, PMID: 37006312 PMC10050748

[ref2] Anton S. D. Moehl K. Donahoo W. T. Marosi K. Lee S. A. Mainous A. G.. (2018). Flipping the metabolic switch: understanding and applying the health benefits of fasting. Obesity 26, 254–268. doi: 10.1002/oby.22065, PMID: 29086496 PMC5783752

[ref3] Dai W. Wang X. Teng H. Li C. Wang B. Wang J. (2019). Celastrol inhibits microglial pyroptosis and attenuates inflammatory reaction in acute spinal cord injury rats. Int. Immunopharmacol. 66, 215–223. doi: 10.1016/j.intimp.2018.11.029 30472522

[ref4] Dai S. Wei J. Zhang H. Luo P. Yang Y. Jiang X.. (2022). Intermittent fasting reduces neuroinflammation in intracerebral hemorrhage through the Sirt3/Nrf2/HO-1 pathway. J. Neuroinflammation 19:122. doi: 10.1186/s12974-022-02474-2, PMID: 35624490 PMC9137193

[ref5] Dimitrijevic M. R. Kakulas B. A. (2020). Spinal cord injuries, human neuropathology and neurophysiology. Acta Myol. 39, 353–358. doi: 10.36185/2532-1900-039 33458591 PMC7783432

[ref6] Duboc H. Taché Y. Hofmann A. F. (2014). The bile acid TGR5 membrane receptor: from basic research to clinical application. Dig. Liver Dis. 46, 302–312. doi: 10.1016/j.dld.2013.10.021, PMID: 24411485 PMC5953190

[ref7] Fann D. Y.-W. Santro T. Manzanero S. Widiapradja A. Cheng Y.-L. Lee S.-Y.. (2014). Intermittent fasting attenuates inflammasome activity in ischemic stroke. Exp. Neurol. 257, 114–119. doi: 10.1016/j.expneurol.2014.04.017, PMID: 24805069

[ref8] Frank J. Gupta A. Osadchiy V. Mayer E. A. (2021). Brain–gut–microbiome interactions and intermittent fasting in obesity. Nutrients 13:584. doi: 10.3390/nu13020584, PMID: 33578763 PMC7916460

[ref9] Ghosh S. Castillo E. Frias E. S. Swanson R. A. (2018). Bioenergetic regulation of microglia. Glia 66, 1200–1212. doi: 10.1002/glia.23271, PMID: 29219210 PMC5903989

[ref10] Gregor A. Panteva V. Bruckberger S. Auñon-Lopez A. Blahova S. Blahova V.. (2024). Energy and macronutrient restriction regulate bile acid homeostasis. J. Nutr. Biochem. 124:109517. doi: 10.1016/j.jnutbio.2023.109517, PMID: 37925090

[ref11] Hadem I. K. H. Majaw T. Kharbuli B. Sharma R. (2019). Beneficial effects of dietary restriction in aging brain. J. Chem. Neuroanat. 95, 123–133. doi: 10.1016/j.jchemneu.2017.10.001 29031555

[ref12] Harris L. Hamilton S. Azevedo L. B. Olajide J. De Brún C. Waller G.. (2018). Intermittent fasting interventions for treatment of overweight and obesity in adults: a systematic review and meta-analysis. JBI Database System Rev. Implement. Rep. 16, 507–547. doi: 10.11124/JBISRIR-2016-003248, PMID: 29419624

[ref13] Hou Y. Luan J. Huang T. Deng T. Li X. Xiao Z.. (2021). Tauroursodeoxycholic acid alleviates secondary injury in spinal cord injury mice by reducing oxidative stress, apoptosis, and inflammatory response. J. Neuroinflammation 18:216. doi: 10.1186/s12974-021-02248-2, PMID: 34544428 PMC8454169

[ref14] Hu X. Yan J. Huang L. Araujo C. Peng J. Gao L.. (2021). INT-777 attenuates NLRP3-ASC inflammasome-mediated neuroinflammation via TGR5/cAMP/PKA signaling pathway after subarachnoid hemorrhage in rats. Brain Behav. Immun. 91, 587–600. doi: 10.1016/j.bbi.2020.09.016 32961266 PMC7749833

[ref15] Hurley M. J. Bates R. Macnaughtan J. Schapira A. H. V. (2022). Bile acids and neurological disease. Pharmacol. Ther. 240:108311. doi: 10.1016/j.pharmthera.2022.108311 36400238

[ref16] Jeong M. Plunet W. Streijger F. Lee J. H. T. Plemel J. R. Park S.. (2011). Intermittent fasting improves functional recovery after rat thoracic contusion spinal cord injury. J. Neurotrauma 28, 479–492. doi: 10.1089/neu.2010.1609, PMID: 21219083 PMC3119327

[ref17] Jiang W. He F. Ding G. Wu J. (2023). Elamipretide reduces pyroptosis and improves functional recovery after spinal cord injury. CNS Neurosci. Ther. 29, 2843–2856. doi: 10.1111/cns.14221 37081763 PMC10493668

[ref18] Jiang W. Li M. He F. Zhou S. Zhu L. (2017). Targeting the NLRP3 inflammasome to attenuate spinal cord injury in mice. J. Neuroinflammation 14:207. doi: 10.1186/s12974-017-0980-9, PMID: 29070054 PMC5657095

[ref19] Kim S. J. Ko W.-K. Jo M.-J. Arai Y. Choi H. Kumar H.. (2018). Anti-inflammatory effect of Tauroursodeoxycholic acid in RAW 264.7 macrophages, bone marrow-derived macrophages, BV2 microglial cells, and spinal cord injury. Sci. Rep. 8:3176. doi: 10.1038/s41598-018-21621-5, PMID: 29453346 PMC5816629

[ref20] Ko W.-K. Kim S. J. Jo M.-J. Choi H. Lee D. Kwon I. K.. (2019). Ursodeoxycholic acid inhibits inflammatory responses and promotes functional recovery after spinal cord injury in rats. Mol. Neurobiol. 56, 267–277. doi: 10.1007/s12035-018-0994-z 29691718

[ref21] Kong X. Yuan Z. Cheng J. (2017). The function of NOD-like receptors in central nervous system diseases. J Neurosci Res 95, 1565–1573. doi: 10.1002/jnr.24004 28029680

[ref22] Kumar R. Lim J. Mekary R. A. Rattani A. Dewan M. C. Sharif S. Y.. (2018). Traumatic spinal injury: global epidemiology and worldwide volume. World Neurosurg. 113, e345–e363. doi: 10.1016/j.wneu.2018.02.033, PMID: 29454115

[ref23] Li M. Yang X. Sun N. Tang R. Wang W. Huang X.. (2022). Dietary restriction may attenuate the expression of cell death–related proteins in rats with acute spinal cord injury. World Neurosurg. 162, e475–e483. doi: 10.1016/j.wneu.2022.03.035 35304344

[ref24] Liang H. Matei N. McBride D. W. Xu Y. Zhou Z. Tang J.. (2021). TGR5 activation attenuates neuroinflammation via Pellino3 inhibition of caspase-8/NLRP3 after middle cerebral artery occlusion in rats. J. Neuroinflammation 18:40. doi: 10.1186/s12974-021-02087-1, PMID: 33531049 PMC7856773

[ref25] Lin X. Zhu X. Xin Y. Zhang P. Xiao Y. He T.. (2023). Intermittent fasting alleviates non-alcoholic Steatohepatitis by regulating bile acid metabolism and promoting fecal bile acid excretion in high-fat and high-cholesterol diet fed mice. Mol. Nut.r Food Res. 67:e2200595. doi: 10.1002/mnfr.202200595, PMID: 37148502

[ref26] Liu Z. Tu K. Zou P. Liao C. Ding R. Huang Z.. (2023). Hesperetin ameliorates spinal cord injury by inhibiting NLRP3 inflammasome activation and pyroptosis through enhancing Nrf2 signaling. Int. Immunopharmacol. 118:110103. doi: 10.1016/j.intimp.2023.110103 37001385

[ref27] Lun W. Yan Q. Guo X. Zhou M. Bai Y. He J.. (2024). Mechanism of action of the bile acid receptor TGR5 in obesity. Acta Pharm. Sin. B 14, 468–491. doi: 10.1016/j.apsb.2023.11.011, PMID: 38322325 PMC10840437

[ref28] Man S. M. Karki R. Kanneganti T. (2017). Molecular mechanisms and functions of pyroptosis, inflammatory caspases and inflammasomes in infectious diseases. Immunol. Rev. 277, 61–75. doi: 10.1111/imr.12534, PMID: 28462526 PMC5416822

[ref29] Mattson M. P. Longo V. D. Harvie M. (2017). Impact of intermittent fasting on health and disease processes. Ageing Res. Rev. 39, 46–58. doi: 10.1016/j.arr.2016.10.005, PMID: 27810402 PMC5411330

[ref30] Mattson M. P. Moehl K. Ghena N. Schmaedick M. Cheng A. (2018). Intermittent metabolic switching, neuroplasticity and brain health. Nat. Rev. Neurosci. 19, 81–94. doi: 10.1038/nrn.2017.156, PMID: 29321682 PMC5913738

[ref31] Monteiro-Cardoso V. F. Corlianò M. Singaraja R. R. (2021). Bile acids: a communication channel in the gut-brain axis. NeuroMolecular Med. 23, 99–117. doi: 10.1007/s12017-020-08625-z, PMID: 33085065

[ref32] Panizza C. E. Lim U. Yonemori K. M. Cassel K. D. Wilkens L. R. Harvie M. N.. (2019). Effects of intermittent energy restriction combined with a Mediterranean diet on reducing visceral adiposity: a randomized active comparator pilot study. Nutrients 11:1386. doi: 10.3390/nu11061386, PMID: 31226790 PMC6627434

[ref33] Patterson R. E. Sears D. D. (2017). Metabolic effects of intermittent fasting. Annu. Rev. Nutr. 37, 371–393. doi: 10.1146/annurev-nutr-071816-064634 28715993 PMC13170603

[ref34] Pereira M. Liang J. Edwards-Hicks J. Meadows A. M. Hinz C. Liggi S.. (2024). Arachidonic acid inhibition of the NLRP3 inflammasome is a mechanism to explain the anti-inflammatory effects of fasting. Cell Rep. 43:113700. doi: 10.1016/j.celrep.2024.113700, PMID: 38265935 PMC10940735

[ref35] Plunet W. T. Lam C. K. Lee J. H. T. Liu J. Tetzlaff W. (2010). Prophylactic dietary restriction may promote functional recovery and increase lifespan after spinal cord injury. Ann. N. Y. Acad. Sci. 1198, E1–E11. doi: 10.1111/j.1749-6632.2010.05564.x, PMID: 20590533

[ref36] Plunet W. T. Streijger F. Lam C. K. Lee J. H. T. Liu J. Tetzlaff W. (2008). Dietary restriction started after spinal cord injury improves functional recovery. Exp. Neurol. 213, 28–35. doi: 10.1016/j.expneurol.2008.04.011 18585708

[ref37] Ramachandran R. Manan A. Kim J. Choi S. (2024). NLRP3 inflammasome: a key player in the pathogenesis of life-style disorders. Exp. Mol. Med. 56, 1488–1500. doi: 10.1038/s12276-024-01261-8, PMID: 38945951 PMC11297159

[ref38] Roome R. B. Vanderluit J. L. (2015). Paw-dragging: a novel, sensitive analysis of the mouse cylinder test. JoVE 98:e52701. doi: 10.3791/52701, PMID: 25993447 PMC4541598

[ref39] Rubovitch V. Pharayra A. Har-Even M. Dvir O. Mattson M. P. Pick C. G. (2019). Dietary energy restriction ameliorates cognitive impairment in a mouse model of traumatic brain injury. J. Mol. Neurosci. 67, 613–621. doi: 10.1007/s12031-019-01271-6, PMID: 30734244 PMC6588397

[ref40] Sayadi J. J. Sayadi L. Satteson E. Chopan M. (2021). Nerve injury and repair in a ketogenic milieu: a systematic review of traumatic injuries to the spinal cord and peripheral nervous tissue. PLoS One 16:e0244244. doi: 10.1371/journal.pone.0244244, PMID: 33395427 PMC7781473

[ref41] Serger E. Luengo-Gutierrez L. Chadwick J. S. Kong G. Zhou L. Crawford G.. (2022). The gut metabolite indole-3 propionate promotes nerve regeneration and repair. Nature 607, 585–592. doi: 10.1038/s41586-022-04884-x, PMID: 35732737

[ref42] Shi J. Zhao Y. Wang K. Shi X. Wang Y. Huang H.. (2015). Cleavage of GSDMD by inflammatory caspases determines pyroptotic cell death. Nature 526, 660–665. doi: 10.1038/nature15514 26375003

[ref43] Smaling A. Romero-Ramírez L. Mey J. (2023). Is TGR5 a therapeutic target for the treatment of spinal cord injury? J. Neurochem. 164, 454–467. doi: 10.1111/jnc.15727 36409000

[ref44] Traba J. Geiger S. S. Kwarteng-Siaw M. Han K. Ra O. H. Siegel R. M.. (2017). Prolonged fasting suppresses mitochondrial NLRP3 inflammasome assembly and activation via SIRT3-mediated activation of superoxide dismutase 2. J. Biol. Chem. 292, 12153–12164. doi: 10.1074/jbc.M117.791715, PMID: 28584055 PMC5519366

[ref45] Traba J. Kwarteng-Siaw M. Okoli T. C. Li J. Huffstutler R. D. Bray A.. (2015). Fasting and refeeding differentially regulate NLRP3 inflammasome activation in human subjects. J. Clin. Invest. 125, 4592–4600. doi: 10.1172/JCI83260, PMID: 26529255 PMC4665779

[ref46] Tsankov B. K. Luchak A. Carr C. Philpott D. J. (2024). The effects of NOD-like receptors on adaptive immune responses. Biom. J. 47:100637. doi: 10.1016/j.bj.2023.100637, PMID: 37541620 PMC10796267

[ref47] Vasconcelos A. R. Yshii L. M. Viel T. A. Buck H. S. Mattson M. P. Scavone C.. (2014). Intermittent fasting attenuates lipopolysaccharide-induced neuroinflammation and memory impairment. J. Neuroinflammation 11:85. doi: 10.1186/1742-2094-11-85, PMID: 24886300 PMC4041059

[ref48] Xu S. Wang D. Tan L. Lu J. (2024). The role of NLRP3 inflammasome in type 2 inflammation related diseases. Autoimmunity 57:2310269. doi: 10.1080/08916934.2024.2310269, PMID: 38332696

[ref49] Xu S. Wang J. Zhong J. Shao M. Jiang J. Song J.. (2021). CD73 alleviates GSDMD-mediated microglia pyroptosis in spinal cord injury through PI3K/AKT/Foxo1 signaling. Clin. Transl. Med. 11:e269. doi: 10.1002/ctm2.269, PMID: 33463071 PMC7774461

[ref50] Ye D. He J. He X. (2024). The role of bile acid receptor TGR5 in regulating inflammatory signalling. Scand. J. Immunol. 99:e13361. doi: 10.1111/sji.13361, PMID: 38307496

[ref51] Zhang F. Deng Y. Wang H. Fu J. Wu G. Duan Z.. (2024). Gut microbiota-mediated ursodeoxycholic acids regulate the inflammation of microglia through TGR5 signaling after MCAO. Brain Behav. Immun. 115, 667–679. doi: 10.1016/j.bbi.2023.11.021, PMID: 37989444

[ref52] Zhang Y. Yang W. Li W. Zhao Y. (2021). NLRP3 Inflammasome: checkpoint connecting innate and adaptive immunity in autoimmune diseases. Front. Immunol. 12:732933. doi: 10.3389/fimmu.2021.732933, PMID: 34707607 PMC8542789

