# Supplementary figures and images for: Every-other-day fasting inhibits pyroptosis while regulating bile acid metabolism and activating TGR5 signaling in spinal cord injury
Source: Front Mol Neurosci. 2024 Sep 12;17:1466125. doi: 10.3389/fnmol.2024.1466125 (PMC11424537; doi:10.3389/fnmol.2024.1466125)

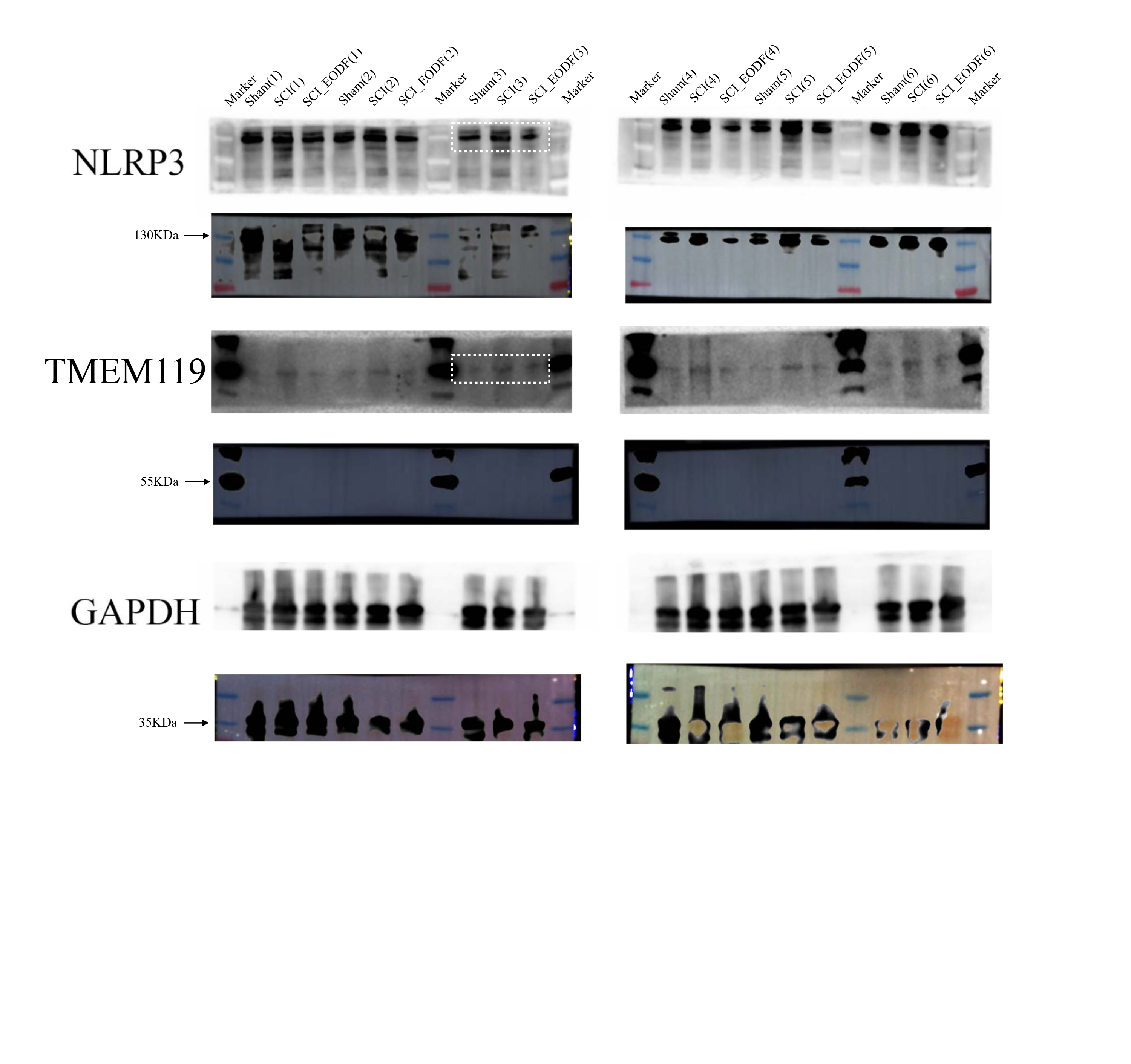

Supplement: Supplementary file 7 [file Image_1.JPEG]

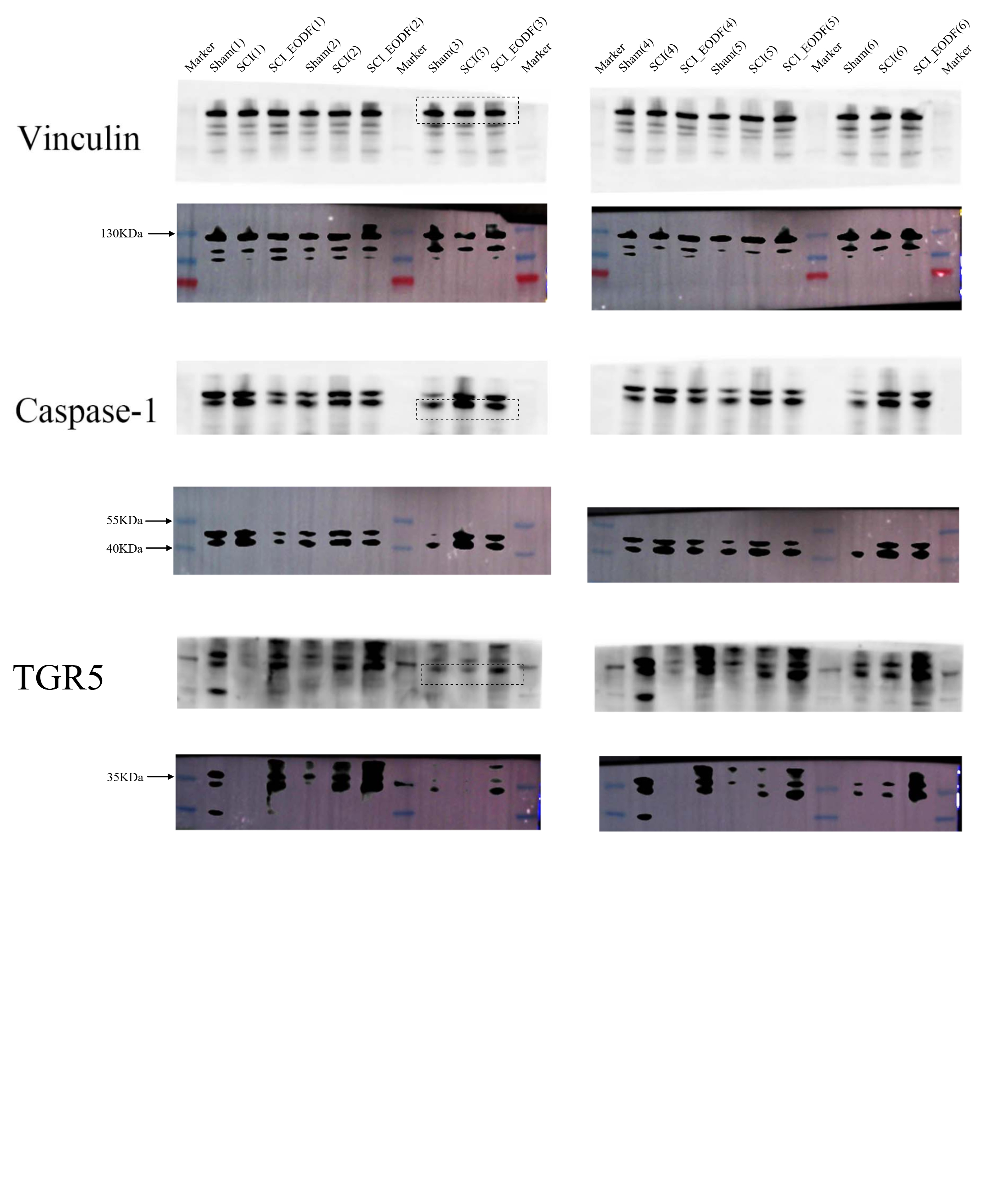

Supplement: Supplementary file 8 [file Image_2.JPEG]

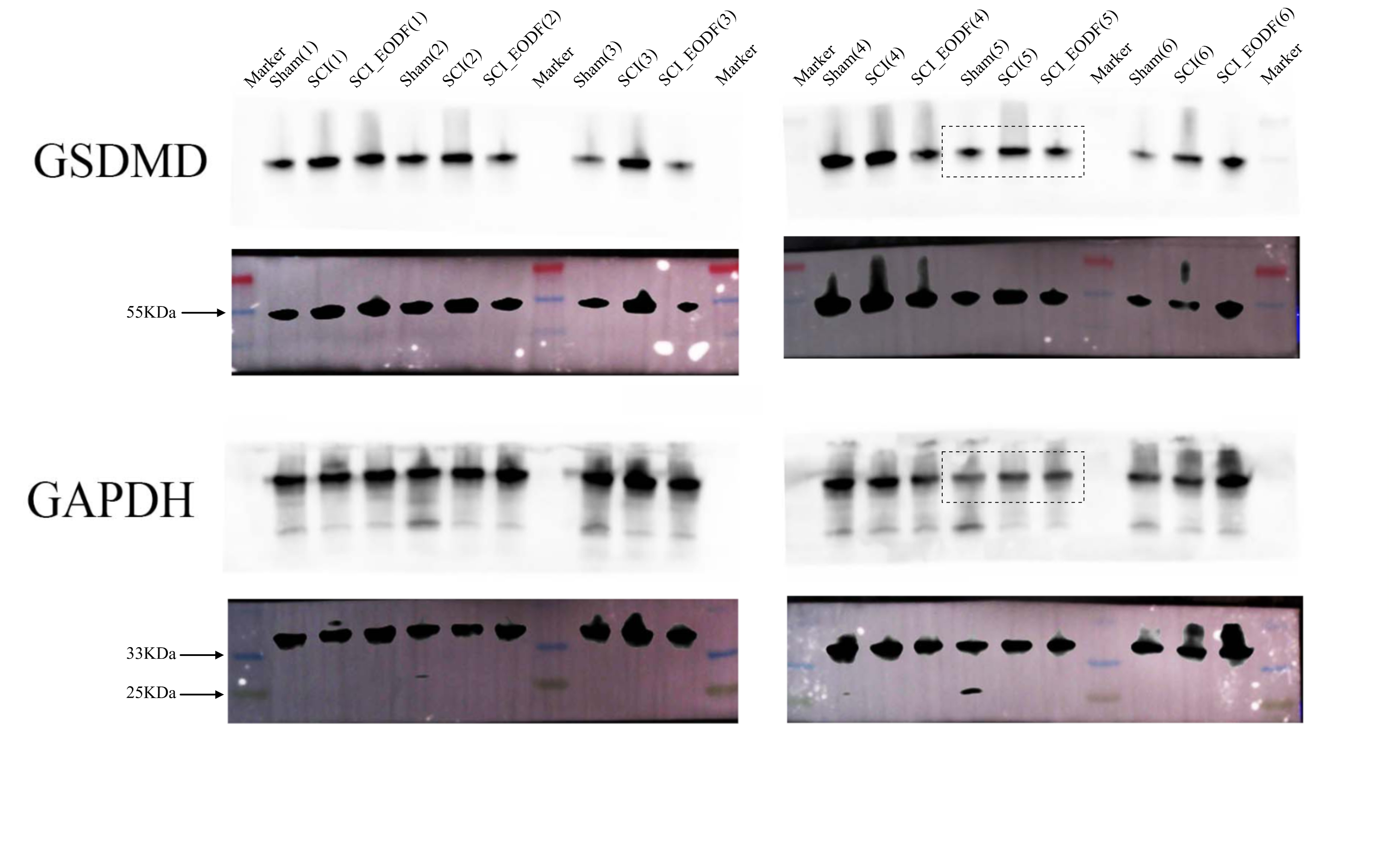

Supplement: Supplementary file 9 [file Image_3.JPEG]
